# Advantages and limitations of navigation‐based multicriteria optimization (MCO) in selectively sparing pharyngeal constrictor muscles in head and neck radiotherapy treatment planning

**DOI:** 10.1002/acm2.70112

**Published:** 2025-06-05

**Authors:** Laura K. Howard, Simon J. P. Meara, Ehab M. Ibrahim, Carl G. Rowbottom

**Affiliations:** ^1^ Medical Physics Department Clatterbridge Cancer Centre NHS Foundation Trust Liverpool UK; ^2^ Department of Physics University of Liverpool Liverpool UK; ^3^ Christie Medical Physics and Engineering The Christie NHS Foundation Trust Manchester UK; ^4^ Division of Cancer Sciences University of Manchester Manchester UK

**Keywords:** head and neck, multicriteria optimization, radiotherapy treatment planning

## Abstract

**Purpose:**

Sparing pharyngeal constrictor muscles (PCMs) during radiotherapy improves patient‐reported swallowing function. This study aimed to explore the feasibility of integrating knowledge‐based planning (KBP) with multicriteria optimization (MCO) in Eclipse v18.0 to selectively spare PCM, quantify the required trade‐off in prophylactic planning target volume (PTV54) coverage, and to evaluate MCO performance.

**Method:**

Ten patients previously planned with KBP for oropharyngeal cancer (65, 60, and 54 Gy in 30 fractions) were retrospectively re‐planned. Clinical plans were further optimized using trade‐off exploration in MCO, with a priority order: spinal cord and brainstem sparing, high‐dose and intermediate‐dose target coverage, PCM sparing, low‐dose target coverage, parotids sparing, remaining organs at risk (OAR). Plans were evaluated based on planning target volumes dose metrics (D_50%_, D_98%_, and D_2%_), homogeneity index (HI), conformity index (CI), and maximum and mean doses to OARs, and paired *t*‐tests were performed. Differences between navigated and deliverable plans were analyzed. One patient underwent 10 identical repeat plan generations.

**Results:**

MCO reduced the average mean dose to the superior and middle PCM, inferior PCM, contralateral parotid, and larynx by 2.0, 3.4, 2.6, and 3.9 Gy, respectively (*p* < 0.05) but at the expense of HI and CI. No difference was observed in average PTV54 D_98%_ between techniques; however, all clinical plans and seven MCO plans achieved D_98%_ ≥ 95%, with three MCO plans modestly compromised (D_98%_ 93.7%–94.6%). Dose metrics between navigated and deliverable plans differed by ≤0.7 Gy for mean doses and ≤1.8 Gy for maximum doses. Pareto surface generation was not repeatable.

**Conclusion:**

MCO effectively balances the trade‐off between PCM sparing and low‐dose target coverage. It may be a valuable tool in the context of personalized care.

## 1 INTRODUCTION

Head and neck (H&N) cancer ranks as the seventh most prevalent cancer worldwide, with over 900 000 new cases and more than 450 000 deaths reported each year.^1^ The primary treatment for locoregionally advanced H&N cancer is either surgery followed by adjuvant radiation therapy (RT), or definitive chemoradiation.^2^ The challenge for RT is achieving local control of the tumor by delivering sufficiently high dose, while avoiding severe and long‐term side effects by limiting critical organ dose; these are inherently contradictory goals. H&N is a complex anatomical site due to the number of radiosensitive organs at risk (OARs) in close proximity to the target; RT is associated with acute and late toxicities that can substantially reduce quality of life.^3^, ^4^ The PARSPORT trial demonstrated that using intensity modulated radiotherapy (IMRT) to spare the parotid glands significantly reduced the incidence of grade 2 or above xerostomia, without compromising locoregional control or overall survival.^5^ The DARS trial showed that dose to the dysphagia and aspiration‐related structures could be reduced using IMRT, improving patient‐reported swallowing function. The authors concluded that sparing of the pharyngeal constrictor muscles (PCMs) should become standard‐of‐care.^6^

Volumetric modulated arc therapy (VMAT) is an extension of IMRT that uses continuous gantry rotation to reduce delivery times and is inversely optimized via minimization of a single cost function. The solution space is a priori unknown; it can be challenging for the oncologist to specify the exact trade‐offs that are desired, especially for OARs in close proximity to, or overlapping, the target. Furthermore, it is challenging to determine whether a more clinically optimal plan could be achieved without time‐consuming re‐optimization. Multicriteria optimization (MCO), an approach that optimizes multiple objectives simultaneously, allows for more personalized treatment plans that balance the trade‐offs between target coverage and OAR sparing. A number of studies have demonstrated significant sparing of H&N OARs with MCO,^7^, ^8^, ^9^, ^10^, ^11^ with two highlighting the potential for PCM sparing with an a priori (lexicographic) approach.^7^, ^10^

The Eclipse treatment planning system (Varian Medical Systems, Palo Alto, CA, USA) takes an a posteriori approach to MCO based on trade‐off exploration. The planner selects N optimization objectives for which they wish to explore trade‐offs, and the optimizer generates a collection of 3N+1 Pareto optimal plans (a plan in which no objective can be improved without degrading at least one other objective) that together form the Pareto surface.^12^ A decision‐maker interacts with a series of sliders, each of which represents an individual trade‐off objective, to navigate to the most clinically favorable plan on the Pareto surface. The fluence pattern in the navigated plan is then converted into a deliverable plan taking into consideration treatment machine limitations, and the final dose is calculated. MCO allows oncologists to directly explore the plan library in real‐time. Muller et al. and Kyroudi et al. used MCO to show that oncologists preferred greater compromise of target coverage for better sparing of the rectum in prostate cancer, as compared to dosimetrists or medical physicists ^13^, ^14^ whilst Xiao et al. showed that oropharyngeal plan navigation by oncologists resulted in better sparing of OARs with high complication rates or overlapping the target.^9^ Oncologist‐driven plan navigation may reduce back‐and‐forth with planners due to this differing prioritization of clinical goals.

RapidPlan is a knowledge‐based planning (KBP) approach within Eclipse. RapidPlan automates treatment planning by estimating achievable OAR dose‐volume histograms (DVHs) for a new patient based on a library of previously treated plans, and automatically generating optimization objectives at the lower bound of the estimated DVH range.^15^ RapidPlan produces highly consistent H&N plans with improved plan quality and increased efficiency.^16^ MCO in Eclipse requires an existing, clinically promising plan (known as the balanced plan) to serve as the approximate center of the Pareto surface.^12^ This limits the solution space to a clinically relevant region. A number of H&N studies have used KBP to generate the balanced plan for subsequent navigation with MCO^17^, ^18^, ^19^; automation ensures consistency and efficiency while MCO individualizes the trade‐offs for the patient. Miguel‐Chumacero et al.^18^ integrated RapidPlan and MCO in three configurations and compared these approaches to RapidPlan and MCO alone, and to VMAT. Trade‐off navigation prioritized reducing the dose to the parotid glands. Adding MCO in any configuration enhanced OAR sparing as compared to VMAT or RapidPlan alone. The greatest parotid sparing was achieved using a RapidPlan model trained on MCO‐navigated plans, followed by further refinement with MCO. However, no dose metrics were reported for other parallel OAR such as PCM. This study combines RapidPlan with MCO to evaluate the feasibility of PCM sparing in oropharyngeal cancer patients, and characterize the level of planning target volume (PTV) compromise required; and explores the usability and reliability of MCO.

## 2 METHODS AND MATERIALS

### 2.1 Patient selection and setup

Eligible patients were those treated in 2022 at our institution, on the definitive (chemo)RT protocol (65, 60, and 54 Gy in 30#) for oropharyngeal squamous cell carcinoma, including bilateral lymph node irradiation, with PCM delineated (*n* = 26). DARS trial constraints for oropharyngeal tumors were requested by the prescribing oncologist. Patients treated off‐protocol (*n* = 1), manually optimized plans (*n* = 3), and plans that achieved the PCM dose constraints (*n* = 9) were excluded. Of the 13 remaining eligible patients, 10 plans were randomly selected for this study. Patients were positioned head‐first supine and immobilized with a 5‐point thermoplastic mask. A CT scan was acquired with 3 mm slices for treatment planning. All patient data were anonymized and approval for this work was granted by the institutional Quality Improvement and Clinical Audit Committee.

### 2.2 Target volume and OAR delineation

The primary tumor and involved lymph nodes were delineated as gross tumor volume (GTV). High (CTV65) and intermediate (CTV60) dose clinical target volumes (CTVs) were delineated according to international consensus guidelines and edited away from natural boundaries (air, uninvolved bone, skin, fascial planes).^20^ A prophylactic dose clinical target volume (CTV54) was delineated to cover at‐risk nodal areas, as per international consensus guidelines.^21^ Geometric expansions of 3–5 mm (PTV65/PTV60) and 5 mm (PTV54) were used to create the PTVs. PTVs were cropped 6 mm from skin surface (unless the CTV extended to the patient surface) and edited to exclude higher‐dose PTVs. Doses of 65, 60, and 54 Gy in 30 fractions, delivered over 42 days, were prescribed to PTV65, PTV60, and PTV54, respectively. Delineated OARs included spinal canal, brainstem, ipsilateral and contralateral parotid glands, larynx, the superior and middle pharyngeal constrictor muscles (SMPCM), and the inferior pharyngeal constrictor muscles (IPCM). 3 mm margins were added to the spinal canal and brainstem to create planning risk volumes (PRVs).

### 2.3 Treatment planning

#### 2.3.1 Planning objectives

Our departmental treatment planning objectives are shown in Table 1. OAR constraints were informed by QUANTEC guidelines,^22^, ^23^, ^24^ and the PARSPORT^5^ and DARS^6^ trials. SMPCM and IPCM were edited to exclude overlap with CTV65 and CTV60, as per DARS. All plans were generated for a Varian TrueBeam (Varian Medical Systems, Palo Alto, CA, USA) linear accelerator with a Millenium 120 multileaf collimator, using 6 MV beams at a dose rate of 600 MU/min. Identical arc configurations were used (two full arcs, collimator 30°/330°, dynamic jaw tracking) and plans were calculated on a 2.5 mm dose grid.

**TABLE 1:** Treatment planning objectives.

Structure	Volume	Constraint^a^
PTV65	D_98%_	>95%
	D_50%_	99%–101%
	D_2%_	<105%
PTV60	D_98%_	>95%
	D_50%_	99%–102%^c^
PTV54	D_98%_	>95%
	D_50%_	99%–102%^c^
Spinal canal	D_0.1cc_	<50 Gy^b^
Brainstem	D_1cc_	<59 Gy^b^
	D_0.1cc_	<54 Gy
Contralateral parotid	D_mean_	<26 Gy
	D_mean_	<14 Gy
Larynx	D_mean_	<50 Gy
	D_mean_	<44 Gy
	V_50Gy_	<27%
SMPCM	D_mean_	<50 Gy
IPCM	D_mean_	<20 Gy

Abbreviations: IPCM, inferior pharyngeal constrictor muscles edited to exclude CTV65 and CTV60; SMCPM, superior and middle pharyngeal constrictor muscles edited to exclude CTV65 and CTV60.

^a^ Percentage of relevant PTV prescribed dose.

^b^ Mandatory dose constraint (applies to PRV).

^c^ Soft upper constraint.

#### 2.3.2 Clinical plans

Clinical VMAT plans were generated with RapidPlan in Eclipse v15.6 using a departmental DVH estimation model, with optimization objectives for SMPCM and IPCM added manually. Details of the RapidPlan model can be found in the Appendix. Planners were able to modify optimization objectives as required to achieve a clinically acceptable plan, aiming to reduce the dose to all delineated OARs to as low as reasonably achievable without compromising PTV65 or PTV60. PTV54 could be compromised to achieve PCM dose constraints, as per DARS.^6^ Optimization build‐up was used if required, and air in PTV was overridden to water for optimization. The photon optimizer (PO) algorithm (v16.6.06) was used for plan optimization and Acuros XB (v16.6.06) used for volume dose calculation. All plans were calculated on a 2.5 mm dose grid. Plans were normalized to PTV65 mean dose, then recalculated with density overrides off.

#### 2.3.3 MCO plans

Planning was performed in Eclipse v18.0. The PO algorithm (v18.0.0) was used for optimization and Acuros XB (v18.0.0) for dose calculation. MCO plans were generated without reference to the dose distribution achieved in the corresponding clinical plan (i.e., the planner did not consider the clinical plan while performing trade‐off exploration). The existing clinical RapidPlan plan served as the balanced plan for MCO optimization; following an upgrade to the departmental version of Eclipse to v18.0, the clinical plan had to be reoptimized using the original optimization objectives. Dose differences were minimal compared to the original clinical plans. The following objectives were selected for trade‐off exploration, and the plan collection generated:

1. PTV65/60/54 minimum and maximum doses;
2. PTV65 median dose;
3. Spinal canal PRV maximum dose;
4. SMPCM and IPCM mean dose;
5. Contralateral and ipsilateral parotid, larynx, oral cavity, and contralateral submandibular gland line objectives.

**FIGURE 1:**
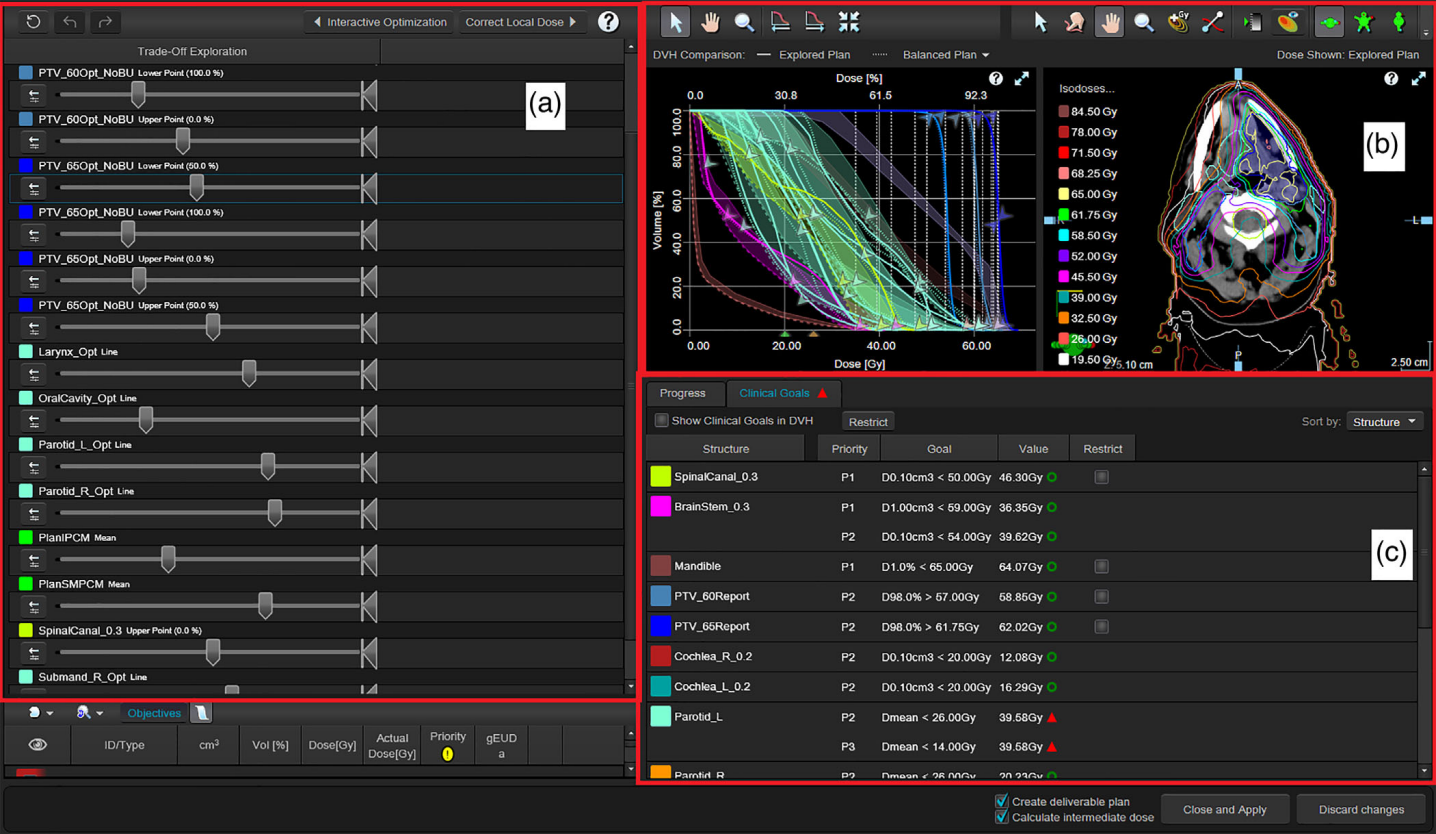
Eclipse v18.0 Trade‐off Exploration workspace. (A) Sliders are available for each objective selected for trade‐off exploration. Moving the slider to the left improves the objective, and sliders can be restricted; (B) dose‐volume histograms and isodose distributions are updated in real‐time during trade‐off exploration; (C) Eclipse Clinical Goals. Spinal canal PRV dose, brainstem PRV dose (if available), PTV65 D_98%_, and PTV60 D_98%_ were restricted at the start of plan navigation by checking the box. If an objective does not have a restrict box, it is either achieved everywhere on the Pareto surface, or nowhere on the Pareto surface. Planning objectives that are within tolerance are shown by green circles and those that are out of tolerance are shown by red triangles. PRV, planning risk volume.

Optimization continued from the balanced plan, which was used as the intermediate dose calculation. Once the plan collection is generated a slider for each trade‐off objective is available, representing the range of achievable doses (see Figure 1). Each slider has a selector and a restrictor. Moving the selector to the left improves the objective (and updates the DVHs, the isodose distribution, and the range on the other sliders in real‐time). This dosimetrically averages between plans in the collection. Sliders and clinical goals defined in the protocol can be restricted, limiting the solution space such that restricted clinical goals cannot be violated. Interactive plan navigation followed the DARS order of priority: spinal canal and brainstem PRV doses, PTV65/PTV60 coverage, SMPCM/IPCM doses, PTV54 coverage, parotids dose, other OARs.^6^ Where the OAR constraints shown in Table 1 could not be achieved, dose was minimized consistent with adequate target coverage. Firstly, the “Restrict” box was checked for spinal canal PRV dose, brainstem PRV dose (if available), PTV65 D_98%_, and PTV60 D_98%_ (in that order); see Figure 1. Next, PTV65 median dose was set to 65 Gy using the slider and the slider restricted, to minimize plan normalization. PTV65 maximum dose was limited using the slider restrictor. Then, PTV54 D_98%_ was set using the slider. SMPCM and ICPM doses were then optimized using their respective sliders, visually inspecting the dose distribution slice‐by‐slice to ensure acceptable coverage of PTV65 and PTV60. Compromise of PTV54 coverage more than 1 cm away from IPCM and SMPCM was minimized. The D_98%_ slider for PTV54 was then restricted to avoid subsequent loss of coverage when optimizing parotid and other OARs. Navigation continued until there was no further range on the sliders or OARs could not be improved without unacceptable degradation of PTVs. Once an acceptable plan was achieved, the navigated plan was converted to a deliverable plan and the final dose was calculated. Plans were normalized to PTV65 mean dose and then the dose was recalculated with density overrides off.

### 2.4 Reliability of MCO

#### 2.4.1 Navigated versus deliverable plans

For each patient, the dose‐volume metrics shown in Table 1 were recorded for both the navigated plan and the deliverable plan prior to normalization, and the difference in dose recorded.

#### 2.4.2 Repeatability in identical repeat runs

A patient with cancer of the base of tongue with bilateral positive lymph nodes was selected for 10 identical repeat runs of plan collection generation using MCO. In the clinical plan, the SMPCM and IPCM mean doses were out of tolerance at 55.1 and 43.3 Gy, respectively. Starting from the original balanced plan each time, the same objectives were selected for trade‐off exploration in each run. In the trade‐off exploration workspace, the restrict box was checked for spinal canal PRV dose, brainstem PRV dose, PTV65 D_98%_, and PTV60 D_98%_ (in that order) and a record made of whether SMPCM and IPCM were available to restrict (i.e., whether dose constraints were achievable on the Pareto surface).

### 2.5 Plan evaluation

Clinical Goals within Eclipse were used to report the dose‐volume metrics listed in Table 1 for each plan. Conformity index (CI) and homogeneity index (HI) were calculated for all PTVs. The formula proposed by van't Riet et al.^25^ was used to determine CI:

(1) $$\mathrm{Conformity}\;\mathrm{Index}\;\left(\mathrm{CI}\right)=\frac{TV_{\mathrm{RI}}}{\mathrm{TV}}\;\;\times \;\frac{TV_{\mathrm{RI}}}{V_{\mathrm{RI}}}$$

where TV_RI_ = target volume covered by the 98% (reference) isodose, TV = target volume; and V_RI_ = volume of the 98% (reference) isodose. CI has a value between 0 and 1 and an ideal value of 1 for a perfectly conformal plan. HI was calculated according to ICRU Report 83^26^:

(2) $$\mathrm{Homogeneity}\;\mathrm{Index}\;\left(\mathrm{HI}\right)=\frac{D_{2\%}-\;D_{98\%}}{D_{50\%}}$$

where D_2%_, D_98%_, and D_50%_ are the dose received by 2%, 98%, and 50% of the PTV, respectively. HI has an ideal value of 0, indicating a perfectly homogeneous dose distribution.

### 2.6 Statistical analysis

The Shapiro–Wilk test indicated no significant departures from normal distribution. Therefore, paired *t*‐tests (two‐tail) were used to compare clinical and MCO plans, with *α* = 0.05. A *p*‐value < 0.05 was considered statistically significant. All statistical analysis was performed in SPSS Statistics (version 29.0.2.0) (IBM, Armonk, NY, USA).

## 3 RESULTS

### 3.1 Patients included

Ten patients were included in this treatment planning feasibility study (five tonsil, four base of tongue, and one soft palate), with mean (± standard deviation) PTV volumes 150.1 ± 93.5 cc (PTV65), 79.8 ± 25.6 cc (PTV60), and 344.6 ± 58.7 cc (PTV54). D_mean_ tolerance for SMPCM was exceeded in one clinical plan, for IPCM in six clinical plans, and both OARs exceeded the dose constraint in three clinical plans.

### 3.2 PTV metrics

Plan normalization was ≤0.5% for MCO plans. A comparison of PTV dose‐volume metrics for MCO versus clinical plans is presented in Table 2. All objectives were achieved for PTV65 and PTV60, except for PTV60 D_98%_, which was not achieved in either plan for patient four, due to a significant volume of air within the PTV. Differences in D_50%_ and D_2%_ for PTV65 were statistically, but not clinically, significant. The difference in mean D_98%_ for PTV54 was not clinically or statistically significant, however, this objective was achieved for all clinical plans but only seven MCO plans (D_98%_ = 50.6–51.1 Gy in the remaining plans). Mean PTV54 D_50%_ increased by 1.4% (*p* = 0.005); one clinical plan and seven MCO plans exceeded the soft upper constraint. CTV54 D_99%_ remained above 51.3 Gy (95%) for all plans.

**TABLE 2:** Comparison of dose‐volume metrics for PTV65, PTV60, PTV54, and CTV54 (clinical plans versus MCO).

Target	Metric (Gy)	Clinical Mean ± SD (95% CI)	MCO Mean ± SD (95% CI)	Difference (*p* value)
PTV65	D_98%_	62.7 ± 0.3 (62.4–62.9)	62.7 ± 0.4 (62.4–62.9)	−0.1% (*p* = 0.73)
	D_50%_	65.1 ± 0.0 (65.1–65.1)	65.2 ± 0.1 (65.1–65.3)	+0.2% (*p* = 0.03)
	D_2%_	66.7 ± 0.3 (66.5–66.9)	67.0 ± 0.5 (66.7–67.4)	+0.6% (*p* = 0.006)
PTV60	D_98%_	58.0 ± 0.8 (57.4–58.5)	57.6 ± 0.7 (57.2–58.1)	−0.6% (*p* = 0.08)
	D_50%_	61.8 ± 0.5 (61.4–62.1)	61.8 ± 0.5 (61.5–62.2)	+0.1% (*p* = 0.79)
	D_2%_	64.5 ± 0.5 (64.1–64.8)	64.5 ± 0.4 (64.2–64.9)	+0.1% (*p* = 0.63)
PTV54	D_98%_	51.8 ± 0.4 (51.5–52.1)	51.7 ± 0.7 (51.2–52.2)	−0.2% (*p* = 0.66)
	D_50%_	54.7 ± 0.5 (54.3–55.0)	55.5 ± 0.5 (55.2–55.8)	+1.4% (*p* = 0.005)
	D_2%_	59.3 ± 1.5 (58.3–60.4)	60.1 ± 1.2 (59.2–60.9)	+1.2% (*p* = 0.006)
CTV54	D_99%_	53.3 ± 0.4 (53.0–53.6)	53.7 ± 0.5 (53.3–54.0)	+0.7% (*p* = 0.05)

Abbreviations: CI, confidence interval; CTV, clinical target volume; PTV, planning target volume; PTV54, PTV receiving 54 Gy; PTV60, PTV receiving 60 Gy; PTV65, PTV receiving 65 Gy; SD, standard deviation.

### 3.3 HI and CI

A comparison of HI and CI for MCO versus clinical plans is shown in Table 3. MCO plans were less homogeneous than the clinical plans; HI increased by 10.5% (*p* = 0.03), 6.1% (*p* = 0.05), and 9.7% (*p* = 0.04) for PTV65, PTV60, and PTV54, respectively. MCO plans were also less conformal than clinical plans, with CI reducing by 6.2% (*p* = 0.003) and 5.8% (*p* < 0.001) for PTV60 and PTV54, respectively. MU increased by 14.4% for MCO (*p* = 0.002).

**TABLE 3:** Conformity index, homogeneity index, and monitor units for PTV65, PTV60, and PTV54 (clinical plans versus MCO).

Parameter	Clinical Mean ± SD (95% CI)	MCO Mean ± SD (95% CI)	Difference (*p* value)
HI (PTV65)	0.061 ± 0.010 (0.054–0.068)	0.067 ± 0.010 (0.060–0.075)	+10.0% (*p* = 0.03)
HI (PTV60)	0.105 ± 0.015 (0.095–0.116)	0.112 ± 0.015 (0.101–0.122)	+6.1% (*p* = 0.05)
HI (PTV54)	0.137 ± 0.028 (0.117–0.157)	0.150 ± 0.022 (0.134–0.166)	+9.7% (*p* = 0.04)
CI (PTV65)	0.799 ± 0.076 (0.745–0.853)	0.804 ± 0.031 (0.782–0.826)	+0.6% (*p* = 0.80)
CI (PTV60|PTV65)	0.795 ± 0.056 (0.755–0.835)	0.746 ± 0.050 (0.710–0.782)	−6.2% (*p* = 0.003)
CI (PTV54|PTV60|PTV65)	0.722 ± 0.037 (0.695–0.748)	0.680 ± 0.040 (0.651–0.708)	−5.8% (*p* < 0.001)
MU	536 ± 23 (521–550)	613 ± 71 (568–657)	+14.4% (*p* = 0.002)

Abbreviations: CI, conformity index; HI, homogeneity index; MU, monitor units.

### 3.4 OAR doses

**FIGURE 2:**
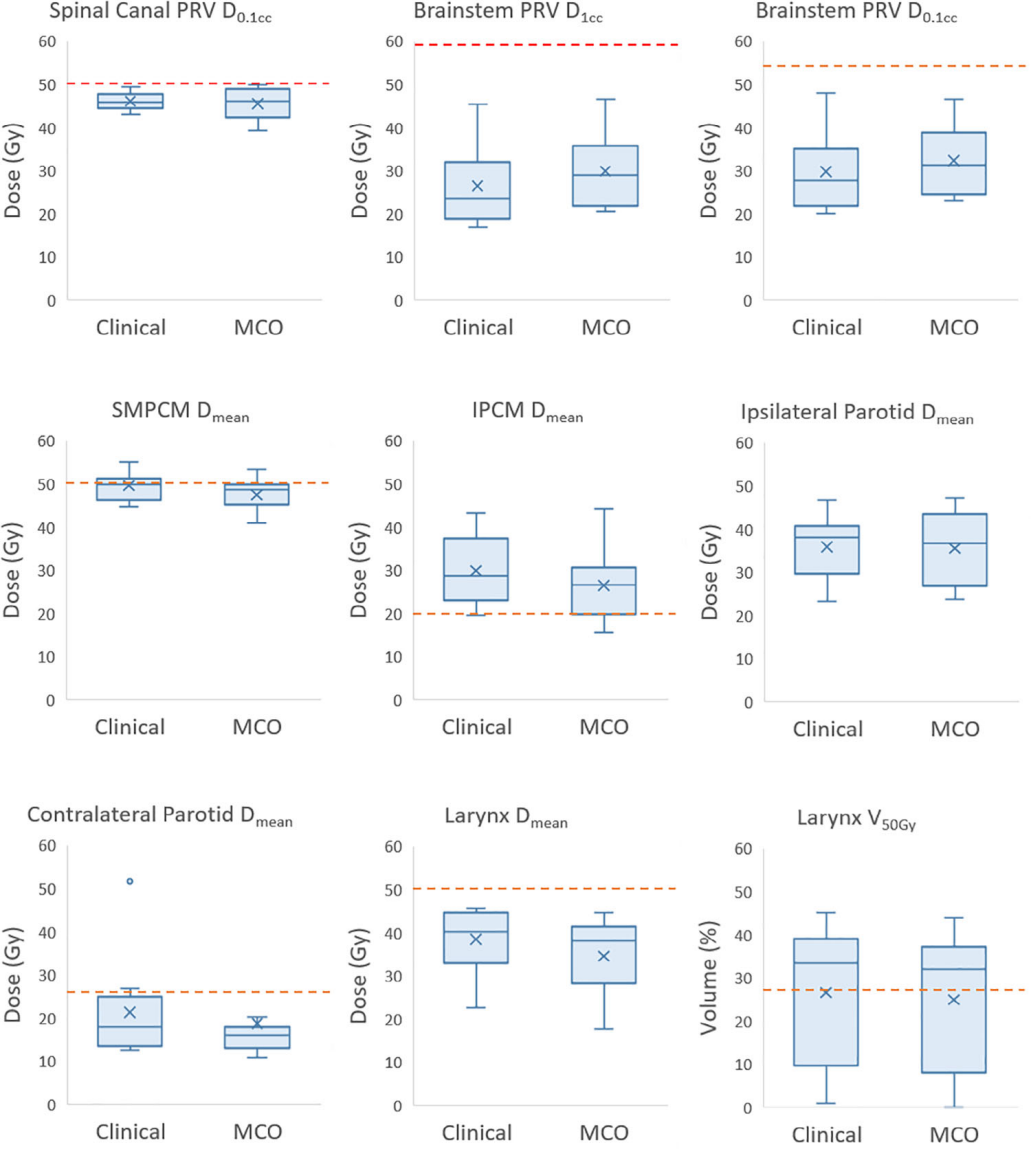
Box plots showing organ‐at‐risk doses for clinical and MCO plans. Red dashed lines indicate mandatory dose constraints; orange dashed lines indicate optimal constraints. IPCM, inferior pharyngeal constrictor muscles edited to exclude CTV65 and CTV60; MCO, multicriteria optimization; oral cavity, oral cavity cropped by 3 mm from PTVs; SMPCM, superior and middle pharyngeal constrictor muscles edited to exclude CTV65 and CTV60.

**FIGURE 3:**
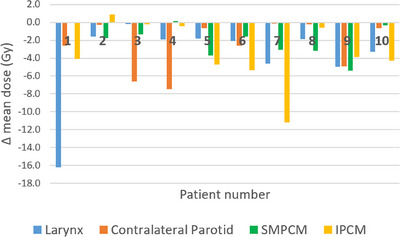
Per‐patient differences in organ‐at‐risk mean doses for MCO versus clinical plans. MCO, multicriteria optimization.

**FIGURE 4:**
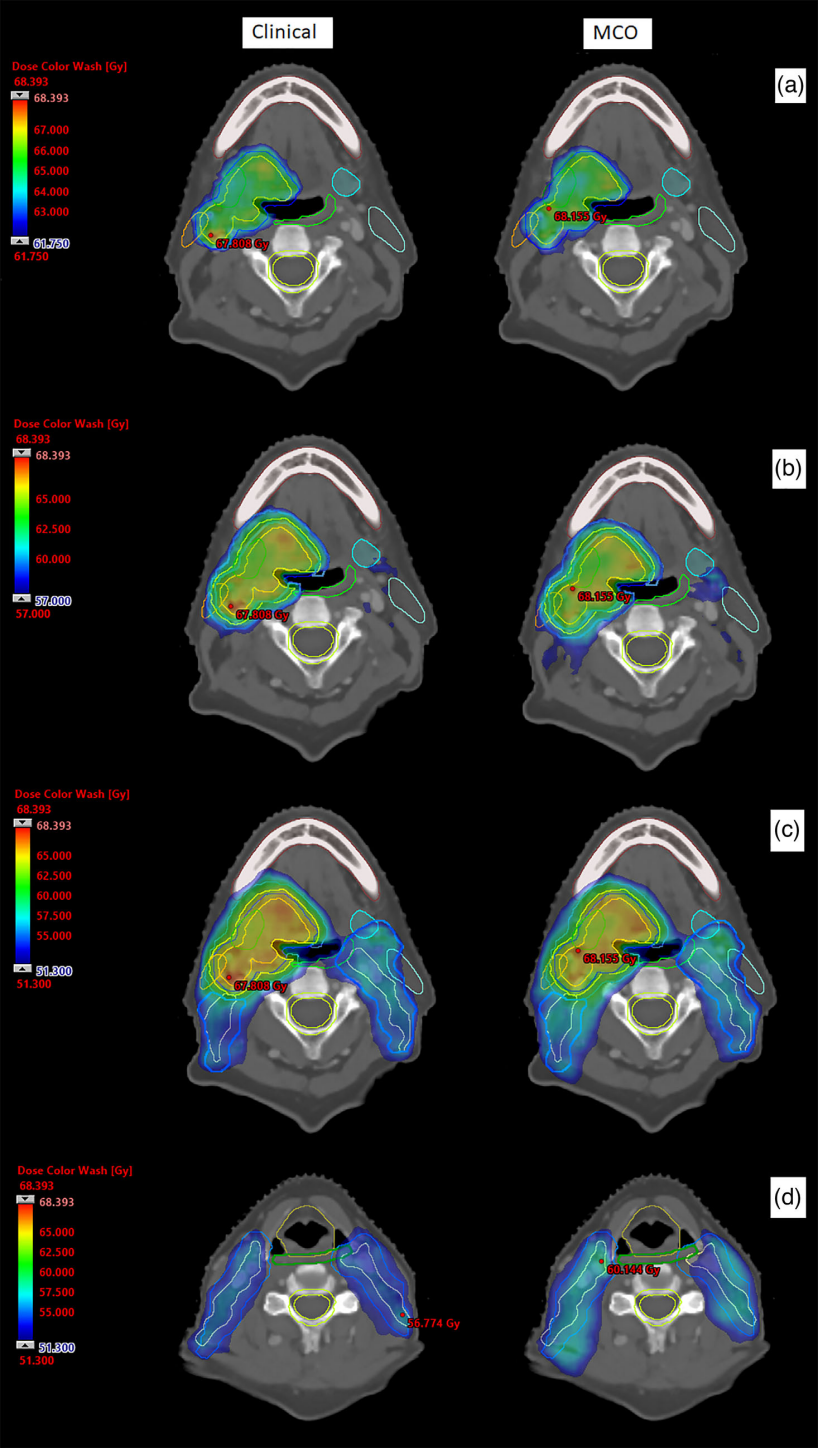
Colorwash dose distribution for patient five, for the clinical plan (left) and MCO plan (right). A 61.75 Gy isodose, PTV65 shown in blue; B 57 Gy isodose, PTV60 shown in steel blue; C 51.3 Gy isodose, PTV54 shown in light blue; D 51.3 Gy isodose, PTV54 shown in light blue. Other structures: SMPCM—green; IPCM—dark green; spinal canal/PRV—yellow green; contralateral parotid—aquamarine; ipsilateral parotid—orange; larynx—olive. Submandibular glands are also shown. MCO, multicriteria optimization; PRV, planning risk volume.

A comparison of OAR doses for MCO versus clinical plans is presented in Figure 2. SMPCM, IPCM, contralateral parotid and larynx mean dose decreased by an average of 2.0 Gy (*p* = 0.006), 3.4 Gy (*p* = 0.01), 2.6 Gy (*p* = 0.02) and 3.9 Gy (*p* = 0.03), respectively. The achievable sparing of these OARs varied widely between patients; see Figure 3. Six clinical and eight MCO plans achieved the SMPCM D_mean_ constraint; one clinical and three MCO plans achieved the IPCM D_mean_ constraint. Mean brainstem PRV D_1cc_ increased by 3.4 Gy (*p* = 0.001) and D_0.1cc_ by 2.6 Gy (*p* = 0.01) for MCO but remained well below tolerance in all cases. A representative dose distribution for patient five is shown in Figure 4.

### 3.5 Navigated versus deliverable plans

**FIGURE 5:**
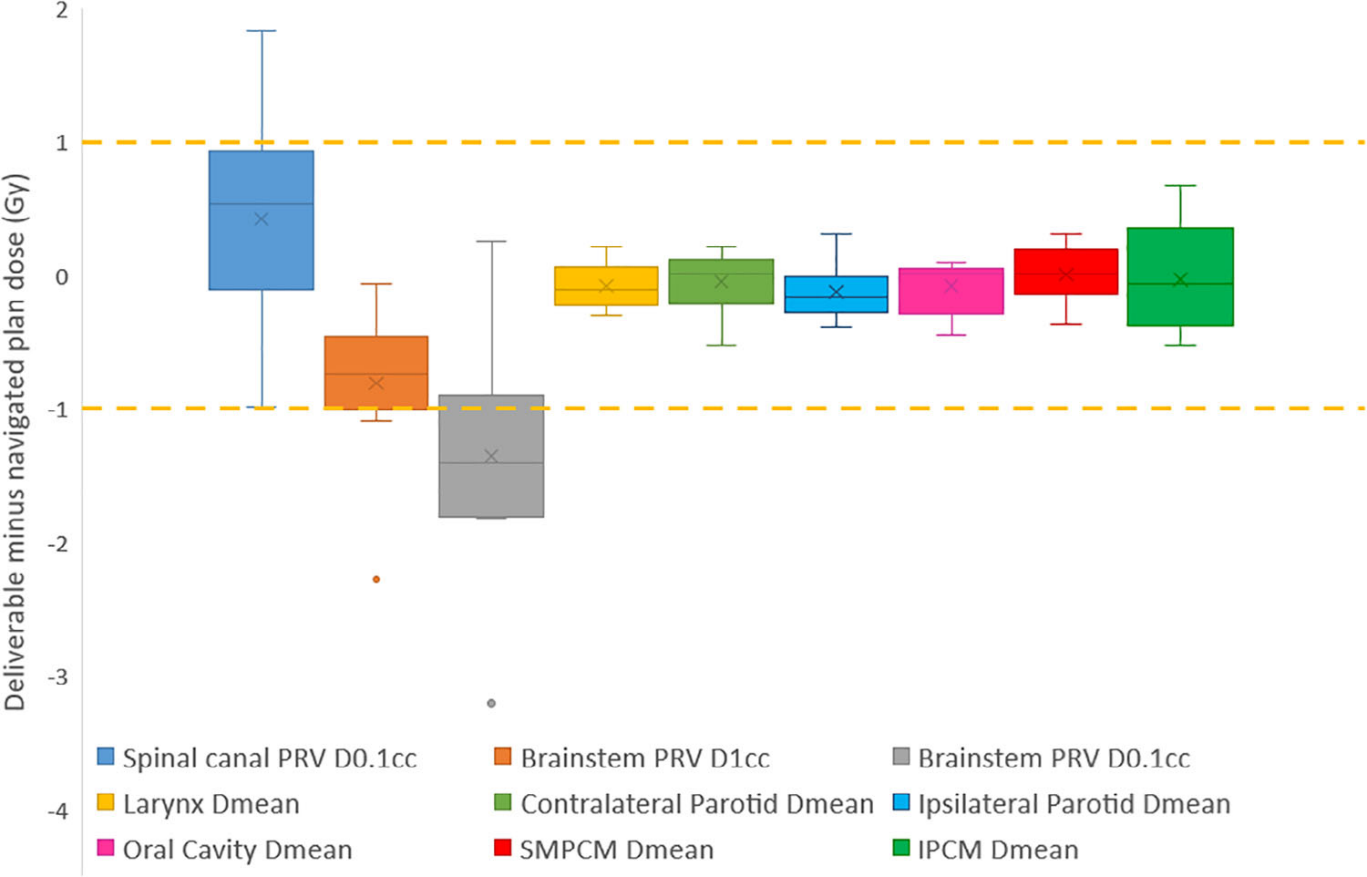
Box‐and‐whisker plot showing the dose difference between MCO deliverable plans and navigated plans, for each organ‐at‐risk. Orange dashed lines indicate ± 1 Gy dose difference. MCO, multicriteria optimization.

The difference between deliverable and navigated plans is presented in Figure 5. Mean dose objectives were within ±0.7 Gy. Differences were more pronounced for maximum dose constraints, with a maximum difference of +1.8 Gy for the spinal canal PRV.

### 3.6 Identical repeat runs

Following restriction of spinal canal PRV dose, brainstem PRV dose, PTV65 D_98%_ and PTV60 D_98%_ using the restrict function in Clinical Goals, the SMPCM mean dose constraint was available to restrict (i.e., the dose constraint was achievable on the Pareto surface) in 7 out of 10 runs, and unavailable to restrict in three runs. The IPCM mean dose constraint was not achievable in any run.

## 4 DISCUSSION

VMAT planning can be a time‐consuming process of multiple optimizations and since there is no solution that simultaneously optimizes each objective, there may be no obvious “best” plan. It can be challenging to articulate the desired trade‐offs precisely without knowing what is feasible. MCO enables dosimetrists or oncologists to explore trade‐offs in real‐time and select the most clinically favorable plan, without reoptimizing and recalculating dose each time. MCO has shown promise in reducing OAR dose for H&N RT across multiple vendors and approaches.^7^, ^8^, ^9^, ^10^, ^11^, ^27^ Sparing the dysphagia and aspiration related structures was shown to improve patient‐reported swallowing function in a recent phase III randomized clinical trial.^6^ This study combined MCO with RapidPlan to evaluate the advantages and limitations of MCO in selectively sparing PCM in patients with oropharyngeal cancer, following a standardized order of priority in the Eclipse v18.0 treatment planning system.

Two studies have explicitly reported PCM sparing with MCO as compared to clinical plans; both used an a priori (lexicographic) approach. In contrast to the a posteriori (navigation‐based) approach in the present study, a single Pareto‐optimal plan was generated, based on lexicographic optimization. All trade‐offs within an a priori assigned ‘wish list’, containing prioritized objective functions and hard constraints which cannot be violated, are balanced within the plan. Voet et al.^7^ reported a mean dose reduction of 3.3 ± 1.1 Gy (maximum 9.2 Gy) for PCM, esophagus and larynx and a 2.4% ± 4.9% (maximum 18.5%) reduction in NTCP for the parotid glands, whilst Biston et al.^10^ reported a mean dose reduction of 2.9 Gy for PCMs and 1.7 Gy for the right parotid in nasopharyngeal cases (but no difference for lower H&N plans). These values are consistent with our study, where MCO reduced the average mean dose to SMPCM (−2.0 Gy), IPCM (−3.4 Gy), contralateral parotid (−2.6 Gy) and larynx (−3.9 Gy), although the achievable sparing varied between patients. Compared to clinical RapidPlan plans, a further two plans achieved the SMPCM constraint, and a further two plans achieved the IPCM constraint. The DARS study^6^ was powered to detect a clinically meaningful 10‐point increase in the MD Anderson Dysphagia Index composite score^28^ at 12 months post‐radiotherapy. Median reductions in mean doses to SMPCM and IPCM of 7.5 and 21.4 Gy, respectively, were achieved. Although the DARS study did not achieve the pre‐specified 10‐point increase in mean MDADI score, a 7.2‐point increase was reported. The dose reductions achieved using MCO in the present study are significantly smaller than those reported in DARS and the clinical impact may therefore be modest. The probability of microscopic disease appears to decrease with increasing distance from the GTV in H&N squamous cell carcinoma,^29^ and the rate of recurrence in low‐risk electively irradiated lymph node regions following definitive RT for H&N cancer is low.^30^ It may therefore be desirable to compromise elective nodal targets to spare OARs. In DARS and the present study, the low dose target could be compromised to spare PCM. MCO was effective at balancing the compromise of OAR sparing and PTV54 coverage proximal to PCM. Kaplan et al. [31] used MCO to explore patient‐specific trade‐offs between peripheral intermediate‐dose target coverage and OAR in three dose‐level H&N RT, concluding that there was considerable variation in what was achievable and per‐patient evaluation is warranted. In our study, there was no mean difference in PTV54 coverage between techniques however seven MCO and 10 clinical plans achieved this constraint. This was an acceptable clinical trade‐off in the DARS trial and was considered acceptable in the present study. CTV54 D_99%_ remained above 95% in all plans, while OAR metrics varied considerably. MU increased by 14.4% for MCO, suggesting increased plan complexity. MCO plans were less conformal and more heterogeneous than clinical plans. This is consistent with Anchineyan et al.,^19^ who found that sparing of the spinal cord and parotid came at the expense of homogeneity (Eclipse v15.5), and Xiao et al.^9^ who reported inferior CI and HI with MCO for oropharyngeal plans (RayStation v4.7) (RaySearch Laboratories AB, Stockholm, Sweden).

Three studies have reported differences between navigated and deliverable plans for MCO in Eclipse. Wong et al.^32^ noted a discrepancy of −2.40% for rectum D_50%_ for MCO prostate planning in v16.1. Gebru et al.^33^ reported more limited achievability of OAR dose than the v16.1 trade‐off exploration workspace suggested for hippocampal avoidance whole‐brain RT, but this is likely related to plan normalization as the authors allowed reduction of PTV dose by up to 4 Gy before normalizing to 95% coverage. Differences between navigated and deliverable plans in the present study were shown to be <1 Gy for mean dose objectives and <2 Gy for maximum dose objectives (v18.0). An increase of 1.8 Gy to the spinal canal PRV during calculation of the deliverable plan for one patient took this OAR beyond tolerance, necessitating a re‐plan. Miguel‐Chumacero et al. reported small discrepancies requiring repeat trade‐off exploration for “a few” H&N patients out of a cohort of 20, in v15.5.^18^ This should be taken into consideration during trade‐off exploration for serial OARs close to tolerance.

Mean plan navigation time was 27 min which is within the published range for H&N MCO. Xiao et al.^9^ reported a median active planning time of 9.3 min and 11.9 min for IMRT plans navigated by dosimetrists and physicians, respectively, whilst Kierkels et al.^8^ required 20 min for IMRT Pareto surface navigation. Krayenbeuhl et al.^16^ reported an effective working time of 116 min, which included definition of VMAT optimization structures. A useful new feature in Eclipse v18.0 is the ability to undo and redo the last slider move, which may reduce plan navigation time compared to published data. Although 15 objectives were selected for trade‐off exploration, only nine sliders were used during plan navigation: PTV minimum dose, PTV65 median dose, SMPCM and IPCM mean dose, and contralateral parotid line objective. Time to generate and navigate the plan collection may be reduced with fewer objectives selected for trade‐off exploration.

RapidPlan produces highly consistent H&N plans with improved plan quality and increased efficiency and does not require oncologist input.^16^ One shortcoming of this DVH‐based approach is its failure to provide spatial context, which may necessitate extra work for planners in cases with atypical geometries. MCO adds the possibility of moving from standard‐of‐care to more personalized care and allows the oncologist to take a more active role. Whether this leads to better decision‐making for individual patients remains to be seen. Cagni et al.^34^ recently demonstrated significant inter‐ and intra‐observer variation in plan quality scores for H&N MCO plans, with agreement between radiation oncologists being no better than between radiation oncologists and medical physicists. However, MCO could facilitate discussions between oncologists and patients about preferred trade‐offs for improved shared decision‐making. One of the main advantages of MCO is giving confidence in whether it is possible to achieve a particular dose constraint to avoid time‐consuming re‐optimizations which may or may not produce the desired plan. For one patient, repeat runs of plan collection generation demonstrated that MCO was not reproducible; therefore, this benefit was not realized, undermining the utility of MCO. A potential reason for this discrepancy is the stochastic (as opposed to deterministic) nature of the PO algorithm in Eclipse. In this study, plan collection generation and navigation averaged 40 min, which is not feasible for all patients with current resources. MCO may be better reserved for complex cases potentially requiring oncologist involvement (e.g., reirradiation). Future work will focus on generating and evaluating a separate RapidPlan model that includes PCMs and comparing performance to MCO.

The main limitation is the small sample size (comparable to other recent MCO planning studies^33^, ^35^) and retrospective nature of the study. A prospective study would allow the same planner to complete the RapidPlan and MCO plans and allow for reporting of patient outcomes to assess the translation of dose reduction into clinical benefit. Whilst the capabilities of MCO have been shown in balancing the trade‐off between PCM sparing and PTV54 coverage, there was no oncologist review of plans to confirm clinical acceptability. This study was evaluated against the RATING framework^36^ and received a score of 95%.

## 5 CONCLUSION

MCO in Eclipse v18.0 is able to reduce dose to PCM in patients with oropharyngeal cancer and is effective at balancing the trade‐off between prophylactic dose PTV coverage and PCM sparing. Dosimetric differences between navigated and deliverable plans are small. MCO was found not to be repeatable in terms of indicating achievable OAR sparing.

## AUTHOR CONTRIBUTIONS

Laura Howard contributed to study design; acquisition, analysis, and interpretation of data; and drafted the manuscript. Simon Meara contributed to study design and critical revision of the manuscript. Ehab Ibrahim contributed to study design and critical revision of the manuscript. Carl Rowbottom contributed to study design and critical revision of the manuscript.

## CONFLICT OF INTEREST STATEMENT

The authors declare no conflicts of interest.

## Supporting information



Supporting Information
