# Supplementary material for: Advantages and limitations of navigation‐based multicriteria optimization (MCO) in selectively sparing pharyngeal constrictor muscles in head and neck radiotherapy treatment planning
Source: J Appl Clin Med Phys. 2025 Jun 5;26(7):e70112. doi: 10.1002/acm2.70112 (PMC12256579; doi:10.1002/acm2.70112)
Supplement: Supplementary file 1 — Supporting Information [file ACM2-26-e70112-s001.docx]

**Appendix**

The RapidPlan model used to generate the balanced plan was developed using 152 previously treated clinical VMAT plans, including oropharynx, nasopharynx and larynx subsites. Outliers were removed.

| **Table 1** Model evaluation statistics for 65Gy/60Gy/54Gy in 30# RapidPlan model | | | | | | |
| --- | --- | --- | --- | --- | --- | --- |
| **Structure** | **Number of training plans** | **R^2^** | | | **MSE** | **Outliers** |
| Brain | 144 | | 0.868 | 0.05 | | 13 |
| Brainstem + 3mm | 134 | | 0.859 | 0.07 | | 59 |
| Left cochlear + 2mm | 137 | | 0.847 | 0.05 | | 9 |
| Right cochlear + 2mm | 139 | | 0.869 | 0.04 | | 14 |
| Larynx* | 128 | | 0.779 | 0.26 | | 7 |
| Mandible + 3mm | 153 | | 0.905 | 0.16 | | 8 |
| Oral cavity* | 148 | | 0.827 | 0.16 | | 2 |
| Left parotid* | 123 | | 0.727 | 0.10 | | 2 |
| Right parotid* | 134 | | 0.796 | 0.11 | | 1 |
| Spinal canal + 3mm | 141 | | 0.653 | 0.18 | | 7 |
| Left submandibular gland* | 88 | | 0.822 | 0.20 | | 4 |
| Right submandibular gland* | 90 | | 0.798 | 0.28 | | 7 |
| ***Cropped back by 3mm from PTVs. R^2^, coefficient of determination; MSE, mean squared error.** | | | | | | |
